# In Vitro Effects of Tea Tree Oil (Melaleuca Alternifolia Essential Oil) and its Principal Component Terpinen-4-ol on Swine Spermatozoa

**DOI:** 10.3390/molecules24061071

**Published:** 2019-03-19

**Authors:** Alberto Elmi, Domenico Ventrella, Francesca Barone, Giacomo Carnevali, Gianfranco Filippini, Annamaria Pisi, Stefania Benvenuti, Maurizio Scozzoli, Maria Laura Bacci

**Affiliations:** 1 Department of Veterinary Medical Sciences, University of Bologna, via Tolara di Sopra 50, 40064 Ozzano dell’Emilia (BO), Italy; alberto.elmi2@unibo.it (A.E.); francesca.barone7@unibo.it (F.B.); giacomo.carnevali2@unibo.it (G.C.); marialaura.bacci@unibo.it (M.L.B.); 2 Unit on Ocular Stem Cell and Translational Research, National Eye Institute (NEI), 9000 Rockville Pike, Bethesda, MD 20892, USA; 3 Department of Agricultural Sciences, University of Bologna, via Fanin 44, 40127 Bologna, Italy; gianfranco.filippini@unibo.it (G.F.); annamaria.pisi@unibo.it (A.P.); 4 Department of Life Sciences, University of Modena and Reggio Emilia, via Giuseppe Campi 103, 41125 Modena, Italy; stefania.benvenuti@unimore.it; 5 APA-CT S.r.l., via Sacco Nicola 22, 47122 Forlì, Italy; maurizio@apabio.it

**Keywords:** tea tree oil, melaleuca alternifolia, terpinen-4-ol, essential oil, swine spermatozoa, toxicity

## Abstract

The growing interest towards essential oils stems from their biological capabilities that include antibacterial and antioxidant effects. Such properties may be extremely useful in the reproductive field; nonetheless essential oils show toxic effects that can lead to cell disruption. The present study aimed to evaluate and compare the effects of tea tree oil (TTO) and its principal component terpinen-4-ol (TER) on the morpho-functional parameters of swine spermatozoa. Experimental samples were prepared by suspending 15 × 10^7^ spermatozoa in 5 mL of medium with different concentrations of the above-mentioned compounds: from 0.2 to 2 mg/mL at an interval of 0.2 for TTO, while TER concentrations were adjusted according to its presence in TTO (41.5%). After 3 h incubation at 16 °C, samples were analyzed for pH, viability, acrosome status, and objective motility. The results highlighted a concentration-dependent effect of TTO with total motility as the most sensitive parameter. TER was better tolerated, and the most sensitive parameters were related to membrane integrity, suggesting a different pattern of interaction. The study confirms the importance of evaluating the effects of natural compounds on spermatozoa before exploiting their beneficial effects. Spermatozoa seem to be good candidates for preliminary toxicological screenings in the light of their peculiar properties.

## 1. Introduction

The need for new and improved protocols for preservation of swine artificial insemination (AI) doses has led researchers toward the study of new active compounds with antibiotic and antioxidant potential [1]. Indeed, up to date, only 1% of porcine AIs around the world are conducted with frozen-thawed semen [2] because of the cellular damage that leads to low fertility in this species [3]. During cryopreservation protocols, antioxidants are necessary to improve cryotolerance [4], but synthetic ones do not always perform well in boars thus, lately, plant based ones have begun to be taken into account [5]. Despite the efforts, liquid phase preservation (16 ± 1 °C) of boar AI doses is still the most common storage technique [3]. In this case, antibiotics are essential to limit bacterial growth [6], as this determines alterations to the midpiece, acrosome, and plasma membrane of the spermatozoa leading to worse quality parameters [7,8]. Moreover, the European Union (Council Directive 90/429/EEC) [9] dictates the use of antibiotics in swine seminal AI doses as mandatory to prevent the spreading of diseases. Nonetheless, it is important to acknowledge that antibiotics added to seminal doses may be potentially dangerous for both animal and human health as they can contribute to the phenomenon of antibiotic-resistance in the swine industry [10]. The scenario prospected by the EU regarding the risks of multi-resistant bacteria calls for ethical discussion regarding how actually necessary are antibiotics and what alternatives can be proposed especially in the zoo-technical world. All of the above leads, again, to the need for new molecules and substances with antioxidant and/or antibacterial capabilities.

Traditional medicine, since the dawn of history, has exploited the knowledge about plants and natural compounds for medical treatments, laying the foundations of modern medicine and pharmacology. The growing interest towards the application of natural compounds, and in particular essential oils (EOs), stems from their potential multi-purpose functional use as antibacterial, antiviral, antifungal, and antioxidant agents [11,12,13,14,15]. The essential oil of *Melaleuca alternifolia,* (Maiden & Betche) Cheel, commonly known as tea tree oil (TTO), is a complex mixture of approximately 100 compounds, produced by the homonymous Australian plant, member of the Myrtaceae family [16,17]. TTO is highly employed in medicine and in the pharmaceutical, food and cosmetic industries due to several biological properties (antimicrobial, antioxidant, antitumor, insecticidal, etc.) [13,18,19]. Currently, the composition of *M. alternifolia* essential oil (terpinen-4-ol type) is regulated by the International Organization of Standardization (ISO) 2004, that sets the cut off value for the 15 main components of the TTO [20,21]. The composition of *M. alternifolia* EO is variable and depending to climate, age of leaves, leaf maceration, and duration of distillation [20]. Generally, the TTO contains numerous monoterpene and sesquiterpene as well as aromatic compounds, which the principal is the terpinen-4-ol (TER), also known for biological activities, especially for anti-bacterial effects [19,20,22,23]. Nonetheless, it has been acknowledged that EOs show toxic activities when applied to different cell populations, including fibroblasts, epithelial cells, monocytes, and neutrophils [12,24]. Only a few data have been reported about spermatozoa and EOs both in humans (*Trachyspermum ammi* [25]; *Thymus mumbianus* [26]) and animals (*Rosmarinus officinalis* [27]). On swine spermatozoa, some studies have already been performed about the effects of *Thymbra capitata* and *Rosmarinus officinalis* EOs [28].

The aim of this study was to evaluate and compare the effects of tea tree oil, and its principal component terpinen-4-ol, on the main morpho-functional parameters of swine spermatozoa, as the first step towards possible application in the reproductive industry.

## 2. Results

### 2.1. Chemical Composition

The chemical composition of the Melaleuca alternifolia EO used in the present study is shown in Table 1. As expected, the main component was terpinen-4-ol (41.49%) followed by other components as γ-terpinene (20.55%), α-terpinene (9.59%) and α-terpineol (4.42%).

### 2.2. Sperm Morpho-Functional Parameters

The descriptive analysis of the effects of TTO and TER on the principal sperm morpho-functional parameters is reported in the supplementary material: Tables S1 and S2.

The analysis of variance showed that both treatments statistically altered the majority of the analyzed parameters: viability (V) (TTO *p* < 0.0001; TER *p* < 0.0001), acrosome reaction (AR) (TTO *p* < 0.0001; TER *p* < 0.0001), total motility (TotM) (TTO *p* < 0.0001; TER *p* = 0.0247) and progressive motility (ProgM) (TTO *p* < 0.0001; TER *p* < 0.0001). The only unaltered parameter was pH (TTO *p* = 0.9969; TER *p* = 0.7908).

**Figure 1:**
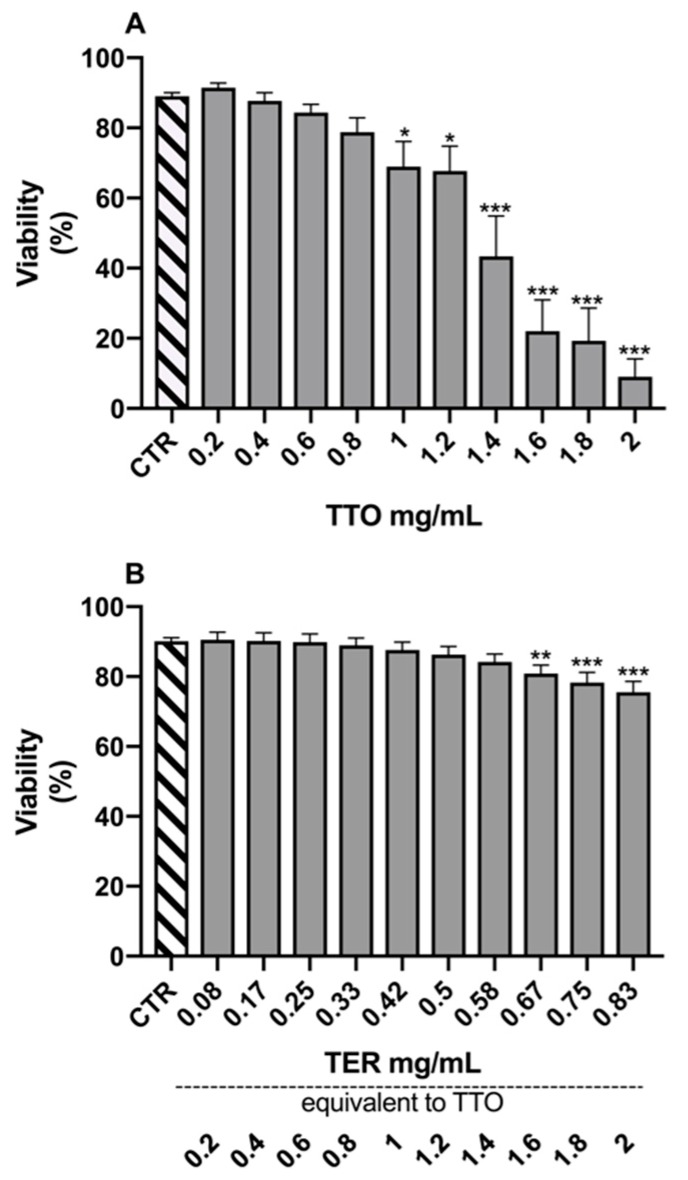
Effects of tea tree oil (**A**) and terpinen-4-ol (**B**) on sperm viability. Data are expressed as mean ± standard error of the mean. CTR = control samples (only emulsifiers). * = *p* < 0.05; ** = *p* < 0.01; *** = *p* < 0.001.

**Figure 2:**
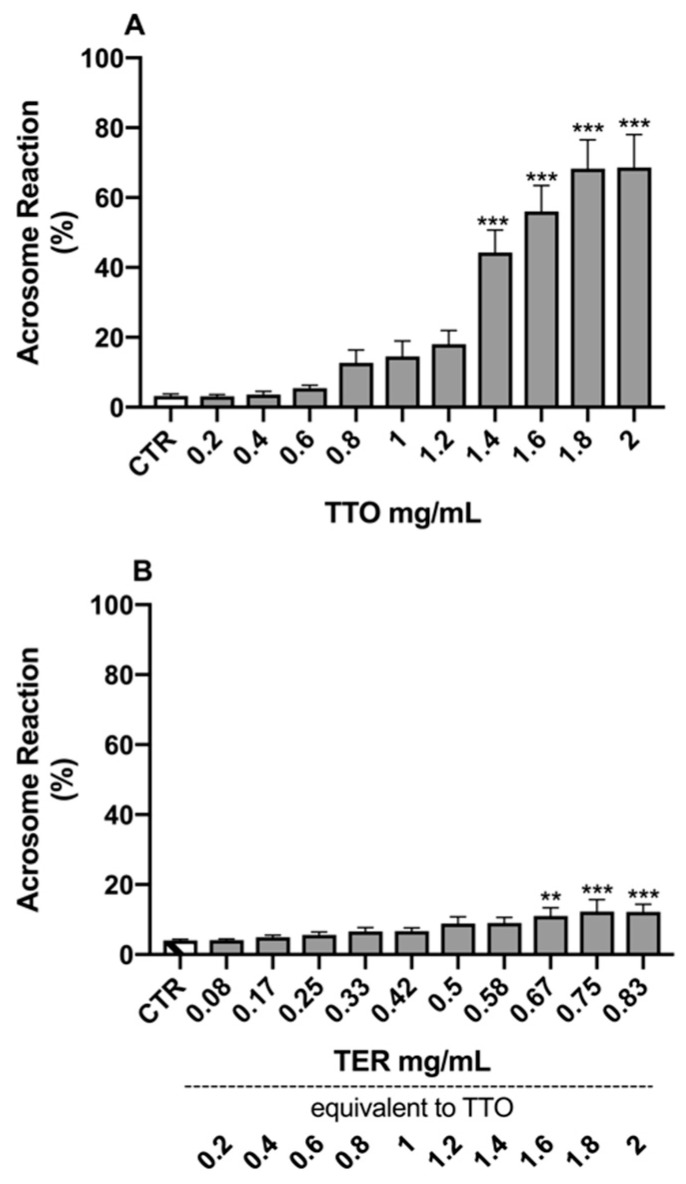
Effects of tea tree oil (**A**) and terpinen-4-ol (**B**) on sperm acrosome reaction. Data are expressed as mean ± standard error of the mean. CTR = control samples (only emulsifiers). ** = *p* < 0.01; *** = *p* < 0.001.

**Figure 3:**
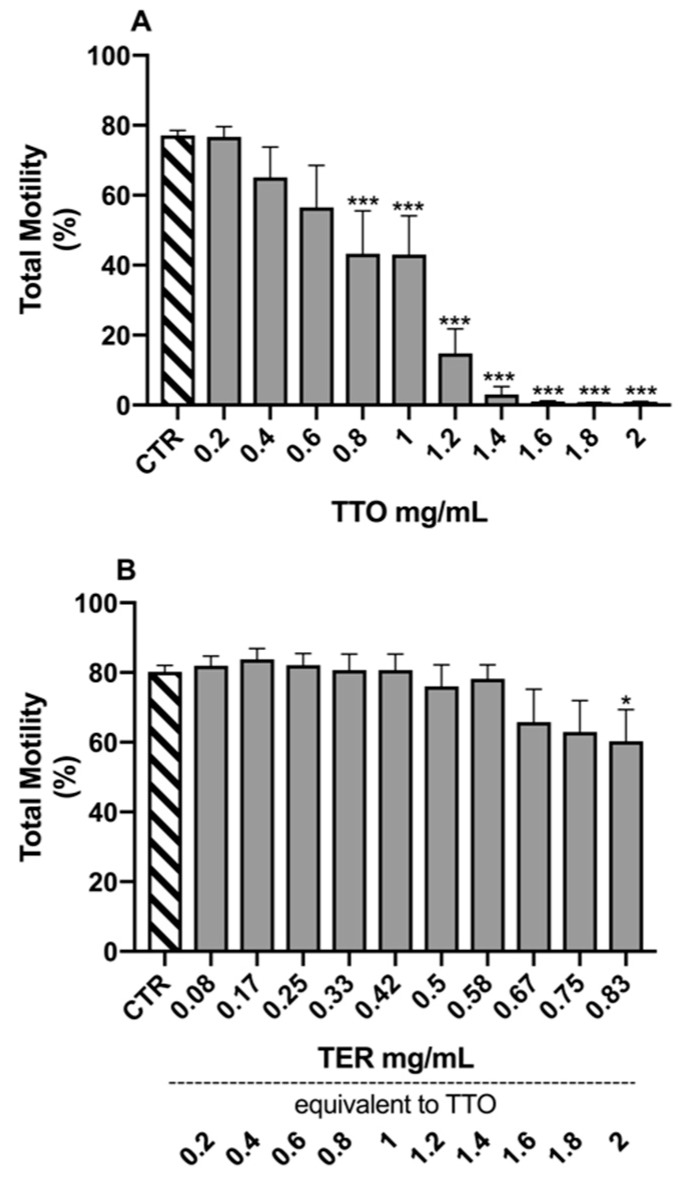
Effects of tea tree oil (**A**) and terpinen-4-ol (**B**) on total sperm motility. Data are expressed as mean ± standard error of the mean. CTR = control samples (only emulsifiers). * = *p* < 0.05; *** = *p* < 0.001.

**Figure 4:**
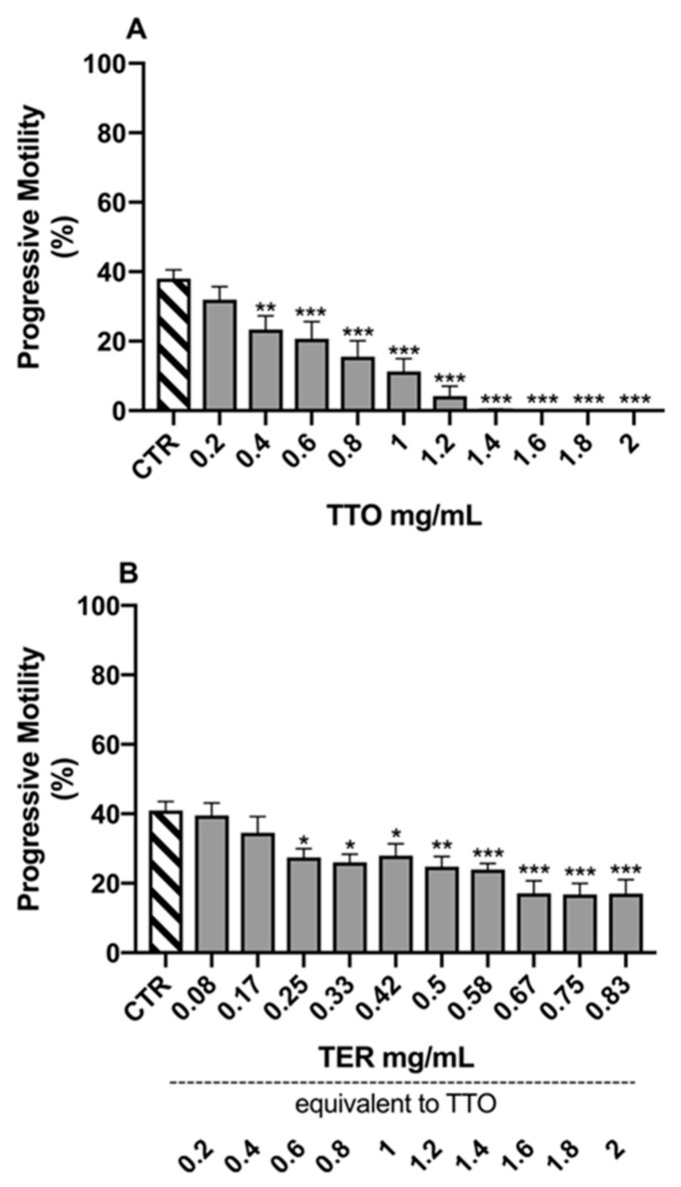
Effects of tea tree oil (**A**) and terpinen-4-ol (**B**) on progressive sperm motility. Data are expressed as mean ± standard error of the mean. CTR = control samples (only emulsifiers). * = *p* < 0.05; ** = *p* < 0.01; *** = *p* < 0.001.

The results of the Dunnett’s tests, comparing the different experimental samples to the control (CTR) for the morpho-functional parameters, are reported in Figure 1, Figure 2, Figure 3 and Figure 4.

The V of spermatozoa treated with TTO (Figure 1A) was statically reduced starting from a concentration of 1 mg/mL (*p* = 0.037) and up to 2 mg/mL (*p* < 0.0001). Spermatozoa treated with TER showed a significant reduction in V (*p* = 0.008; Figure 1B) only for the three highest tested concentrations (equivalent to 1.6, 1.8, and 2 mg/mL of TTO).

Regarding AR, the results showed a statistical increase starting from 1.4 mg/mL for TTO (*p* < 0.0001; Figure 2A) and from 0.67 mg/mL for TER, equivalent to 1.6 mg/mL or TTO (*p* = 0.0026; Figure 2B).

Spermatozoa treated with TTO showed a decreasing trend for TotM (Figure 3A) already at 0.4 mg/mL, statistically appreciable only from 0.8 mg/mL (*p* = 0.0003). The treatment with TER (Figure 3B) did not determine significant alterations of sperm motility, with the only exception for the highest tested concentration of 0.83 mg/mL (*p* = 0.05), corresponding to 2 mg/mL of TTO.

On the other hand, the ProgM showed a slightly different behavior, with statistically significant alterations already starting from 0.4 mg/mL for TTO (*p* = 0.003; Figure 4A) and from 0.25 mg/mL for TER (*p* = 0.043; Figure 4B), equivalent to 0.6 mg/mL of TTO. 

Descriptive and inferential statistics of kinematics parameters are reported in Table 2 (TTO) and Table 3 (TER). The analysis of variance for the TTO group highlighted statistical differences for VAP, VCL, VSL, DAP, DCL, DSL, and STR; on the other hand, only VSL, DSL, LIN, and STR resulted statistically altered for the TER group.

The angular coefficients (β) resulting from the linear regression models between concentrations and morpho-functional semen parameters are reported in Table 4.

### 2.3. Sperm Morphological Evaluation by Scanning Electron Microscopy 

**Figure 5:**
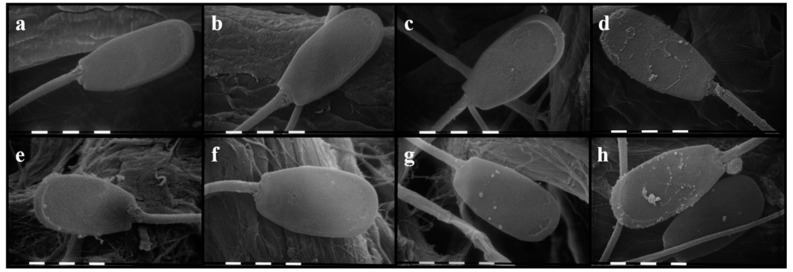
Scanning electron micrographs for the effects of the lowest, middle, and highest concentrations of tea tree oil (TTO) and erpinen-4-ol (TER) on sperm morphology. (**a**) control sample; (**b**) TTO 0.2 mg/mL; (**c**) TTO 1 mg/mL); (**d**) TTO 2 mg/mL; (**e**) control sample; (**f**) TER 0.08 mg/mL; (**g**) TER 0.42 mg/mL; (**h**) TER 0.83 mg/mL.

Images from scanning electron microscopy (SEM) are shown in Figure 5. Samples treated with the lowest concentrations of both substances (TTO Figure 5b; TER Figure 5f) did not show any morphological differences when compared to the control samples (Figure 5a,e), displaying a smooth and intact surface. On the other hand, the effects of the highest concentrations were easily appreciable: 2 mg/mL of TTO (Figure 5d) caused vesiculation, vacuolation, and lyses of membranes throughout the entire cell, 0.83 mg/mL of TER (Figure 5h) determined similar alteration of membranes, but mainly localized in the head region. The middle dosages (Figure 5c,g) determined milder alteration but with the same patterns.

## 3. Discussion

The present study aimed at the evaluation and comparison of the effects of tea tree oil and its main constituent, terpinen-4-ol, on porcine spermatozoa, using the main sperm morpho-functional parameters as study variables. Nowadays, it is common opinion that natural substances, like EOs and or phytoextracts can represent an improvement of the standard cryopreservation protocols of swine AI, but their deleterious potential has to be taken into account and investigated in depth. Indeed, the antibacterial effects of essential oils have to be imputed to a mechanism of interaction with membranes, as highly lipophilic, that may not be selective towards bacteria, as opposed to common antibiotics [12,13]. They can also impair mitochondrial activity by means of membrane depolarization [12,13], potentially leading to the loss of one of the most vital characteristics of spermatozoa represented by motility. This is why the characterization of the direct effects of each essential oil on the spermatic cell is necessary before considering their applications in the reproductive field. Moreover, the peculiar physiology of the spermatozoa, capable of generating motion, makes it a potentially useful tool for the in vitro characterization of general toxicity of exogenous compounds [29,30]. 

*Melaleuca alternifolia* EO (TTO) is defined by the international standard ISO-4730 that specifies certain characteristics of EO in order to facilitate assessment of its quality [21]. In this case, the used EO conforms to the ISO system: 1,8-cineol represents less than 15% (2.15%) and terpinen-4-ol, the main component, more than 30% (41.49%) of the overall composition. 

Overall, TTO seems to be well tolerated by porcine spermatozoa up to a concentration of 0.6 mg/mL; higher quantities of this EO determined increasing impairment in a concentration-dependent manner as shown by the results of the linear regression models for all test parameters excluding pH. Such a pattern of concentration-dependent effects is in agreement with what has already been reported for two other essential oils (*R. officinalis* and *T. capitata*) [28]. Total motility, as already described by literature [27,28,31,32], was the most sensitive thus the limiting parameter with an early reduction, for TTO-treated samples, already at 0.4 mg/mL that became statistically significant at 0.8 mg/mL. On the other hand, membrane integrity seems to be less sensitive to the action of the EO as shown by the results of viability and acrosome status, and ichnographically confirmed by scanning electron microscopy. These findings may suggest an early functional impairment, probably due to interactions with the mitochondrial capability of producing motion [30,33], followed by a morphological one at higher concentrations. This scenario strengthens the necessity to test the toxic effects of exogenous compounds directly in spermatozoa, when the aim is to use them in the reproductive field, as motility impairment cannot be assessed on other cells. The overall findings regarding the effects of the essential oil of *Melaleuca alternifolia* are similar to the ones reported for *Rosmarinus officinalis* in porcine [28] and rooster spermatozoa [27]. 

The rationale for testing terpinen-4-ol by itself was driven by its strong presence in the used EO, accountable for the 41.49% of its whole composition. Indeed, the used concentrations of terpinen-4-ol were calculated on the ones chosen for the EO in order to be able to compare the results between the two groups. Overall, this molecule was better tolerated, as the first toxic effects started to appear at 0.67 mg/mL, the third highest tested concentration. The damage pattern seems opposite to the one determined by TTO, as the most sensitive parameters were, in this case, viability and acrosome reaction. Motility indeed was only significantly altered at the highest concentration, despite a decreasing trend starting from 0.67 mg/mL. Overall, all the tested parameters were less altered upon co-incubation with the single compound, as confirmed by SEM images, in contrast to that described by Hammer et al. [34], who stated that the single compounds of the *Melaleuca alternifolia* essential oil have higher cytotoxic activity when compared to the whole oil. 

Several studies have already reported how terpinen-4-ol by itself still shows antibacterial activity [20], a feature that could be highly exploitable, for example, in swine AI. Nonetheless, the MICs (Minimal Inhibitory Concentrations) reported by literature against different bacteria are highly variable going from 0.6 up to 2.5 mg/mL [35], thus potentially toxic on the basis of the present study. The same statement seems to be true also for TTO, since the MICs available in the literature vary from very low concentrations (0.12–0.5 mg/mL [36]) up to extremely high ones (20 mg/mL [37]. Nonetheless, due to the singular nature of each batch of each essential oil, influenced by a wide variety of agricultural and industrial factors, the analysis of antibacterial capabilities through MICs can be very challenging. It is still important to acknowledge that synergistic effects of lower doses of different molecules, partially already confirmed [38], have to be explored and how recent studies have reported that sub-lethal doses of essential oils and natural compounds may still be effective in controlling bacteria by alteration of peculiar virulence factors [39]. In such a scenario, if the aim is to exploit TTO and/or TER as antimicrobial agents in AI doses, further studies have to be performed to confirm such a possibility.

The results regarding toxicity upon membrane damage are also important since EOs, whether used as potential antimicrobial or spermicidal agents from an industrial point of view, should also be tested for their effect on the female reproductive apparatus. This context is even more delicate and complex to analyze as in vitro testing would not be sufficient. Indeed, the effects exerted by EOs on isolated cell will most likely change in in vivo conditions, where physiological defense mechanisms, such as mucus, are available. 

For both tested substances, progressive motility follows the trend of total motility despite some “earlier” significant differences. Indeed, ProgM, is significantly altered already at “lower” concentrations. Nonetheless, from a biological point of view, these slight discrepancies between TotM and ProgM are only relatively important, as the actual number of progressive motile spermatozoa is directly related to the overall motile ones (ProgM is a small population of TotM). 

The discussion regarding kinematic parameters can be quite difficult, as up to date, their biological meaning and correlation to semen quality and fertility still have to be definitively unveiled. TTO seems to alter velocity (VAP, VCL, and VSL) and distance (DAP, DCL, and DSL) parameters only when used at the highest studied concentration for kinematics; higher concentrations were not evaluated as TotM was not sufficiently relevant. These findings seem to suggest a quantitative impairment in speed, thus in distance, without alteration of the quality of movement as confirmed by the non-altered results of ALH, WOB, and BCF. Indeed, hyper-activated sperm subpopulation, showing abnormal movements, are characterized by increased ALH [33]. The same pattern of velocity–distance alteration was noticed starting from 0.42 mg/mL of TER; corresponding to 1 mg/mL of TTO, but only regarding the straight-line parameters (VSL and DCL). This is particularly interesting as TER-treated samples seem to maintain good TotM (*p* > 0.05) also at higher concentrations then the ones with kinematics alterations. Supposedly, modifications in kinematics parameters with maintained TotM may suggest early impairment, still allowing the spermatozoa to move but with less intensity. Overall, despite the relative lack of literature, different authors have reported how kinematics parameters, especially velocity-related ones, seem to have a good predictive potential when it comes to fertility [40,41]. This is why CASA analyses play a pivotal role when evaluating interactions between new molecules with potential in the reproductive field and spermatozoa [29,30].

The results of the present study suggest that the toxic effects of the used EO are either to be imputed to other constituents rather than terpinen-4-ol or to the synergic interaction of all the components of the oil itself. This point is extremely critical when it comes to the application of phytocompounds and the study of their mechanism of action as the difference in chemotype may alter the outcome of the hypothesized synergistic capabilities of the single components.

## 4. Materials and Methods 

All reagents, unless otherwise specified, were purchased from Sigma-Aldrich (Saint Louis, MO, USA). Essential oil of *Melaleuca alternifolia* (TTO) used for the experiment was provided by APACT (Forlì, FC, Italy). For the experiment, the TTO was reconstituted in 0.5% dimethylsulfoxide (DMSO) and Tween 80 (0.002%) [14]. Terpinen-4-ol was purchased by Moellhausen (Vimercate, MB, Italy).

### 4.1. Chemo-Characterization of the M. alternifolia EO

#### 4.1.1. Gas Chromatography-Mass Detector (GC-MS) Analysis

Analyses were performed on a 7890A gas chromatograph coupled with a 5975C network mass spectrometer (Agilent Technologies, Waldbronn, DE). Compounds were separated on Agilent Technologies HP-5 MS cross-linked poly-5% diphenyl-95% dimethyl polysiloxane (30 m × 0.25 mm i.d., 0.25 mm film thickness) capillary column. The column temperature was initially set at 45 °C, then increased at a rate of 2 °C/min up to 100 °C, then raised to 250 °C at a rate of 5 °C/min, and finally held for 5 min. The injection volume was 0.1 µL, with a split ratio 1:20. Helium was used as the carrier gas, at a flow rate of 0.7 mL/min. The injector, transfer line and ion-source temperature was 250 °C, 280 °C, and 230 °C, respectively. MS detection was performed with electron ionization (EI) at 70 eV, operating in the full-scan acquisition mode in the *m*/*z* range 40–400. EOs were diluted 1:20 (*v*/*v*) with n-hexane before GC-MS analysis.

#### 4.1.2. Gas Chromatography-Flame Ionization Detector (GC-FID) Analysis 

Analyses were carried out on an Agilent Technologies 7820 gas chromatograph (Waldbronn, DE) with a flame ionization detector (FID). Compounds were separated by means of Agilent Technologies HP-5 crosslinked poly-5% diphenyl-95% dimethyl polysiloxane (30 m × 0.32 mm i.d., 0.25 mm film thickness) capillary column. The temperature program was the same as described above. The injection volume was 0.1 µL in split mode 1:20. Helium was used as the carrier gas at a flow rate of 1.0 mL/min. The injector and detector temperature was set at 250 °C, 280 °C, and 230 °C, respectively. EOs and the reference standards were diluted 1:20 (*v*/*v*) with n-hexane before GC-FID analysis. The analyses were performed in duplicate.

#### 4.1.3. Qualitative and Semi-Quantitative Analysis

Compounds were identified by comparing the retention times of the chromatographic peaks with those of authentic reference standards run under the same conditions and by comparing the linear retention indices (LRIs) relative to C8–C40 n-alkanes obtained on the HP-5 column under the above-mentioned conditions with the literature ref. [42]. Peak enrichment by co-injection with authentic reference compounds was also carried out. Comparison of the MS-fragmentation pattern of the target analytes with those of pure components was performed. A mass-spectrum database search was carried out by using the National Institute of Standards and Technology (NIST, Gaithersburg, MD, USA) mass-spectral database (version 2.0d, 2005). Semi-quantification was calculated as the relative percentage amount of each analyte; in particular, the values were expressed as the percentage peak area relative to the total composition of EO obtained by GC-FID analysis.

### 4.2. Boars and Ejaculates 

Three adult boars (Large White × Duroc) housed in single pens, according to the National Law (D.lgs n. 122/2011) in compliance with good practice for animal welfare, were enrolled as ejaculate donors in the present work. Routinely, semen was collected twice a week by an experienced operator using the hand-gloved technique in a pre-heated (37 °C) thermos. 

Eighteen sperm rich fractions (SRFs), six from each boar, were used for this work. Upon collection, the SFR was immediately diluted 1:1 *v*/*v* with an in-house prepared extender (Swine Fertilization Medium, SFM) as previously described [43] without any antibiotics.

To assess overall quality, each SFR was evaluated for spermatozoa concentration by a Thoma haemocytometer chamber, viability (V) by eosin-nigrosine staining, and total motility (TotM) by CASA following the later described protocols [28]. Only SFR with V >85% and TotM >80% were used for the experimental protocol. 

### 4.3. Experimental Protocol

The experimental protocol was performed as previously described by the authors [28].

The TTO and TER were individually tested on three ejaculates from each boar (ejaculates N = 18; TTO *n* = 9; TER *n* = 9). 

For each trial, 11 experimental samples were prepared by suspending 15 × 10^7^ spermatozoa in 5 mL of SFM, with a final concentration of 3 × 10^7^ spermatozoa/mL. Ten samples were added with increasing concentrations of either TTO or TER while one sample, the control (CTR), was only added with emulsifier (DMSO—0.5% and Tween 80—0.2% [14]). The TTO tested concentrations were from 0.2 to 2 mg/mL, at an interval of 0.2; the TER concentrations were adjusted according to the percentage of TER in the used TTO (41, 5%) for each concentration used (Table 5).

Upon preparation, samples were incubated at 16 °C (± 1 °C) in a refrigerated bath for 3 h and subsequently evaluated for the principal morpho-functional parameters.

### 4.4. Evaluation of Spermatozoa Morpho-Functional Parameters

Viability (V) was assessed using Eosin-Nigrosin staining [44], while the percentage of reacted acrosomes (AR) was calculated by means of a modified Coomassie Blue staining protocol [7,31]. The objective analysis of motility, including total (TotM) and progressive (ProgM) motility, was performed by a CASA unit (Hamilton Thorne CEROS II, Animal Motility II, Software version 1.9, Beverly, MA, USA) with a heated stage, after 5 min of incubation of the samples at 37 °C. Since the kinematics parameters can only be appreciated on motile spermatozoa, only samples with TotM ≥20% were used for their analysis as previously done [28]. Analyzed parameters included: average path velocity (VAP), curvilinear velocity (VCL), straight line velocity (VSL), distance of the average path (DAP), curvilinear distance (DCL), straight line distance (DSL), linearity (LIN, calculated from VSL/VCL), straightness (STR; calculated from VSL/VAP), wobble (WOB; calculated from VAP/VCL), amplitude of lateral head displacement (ALH), and beat cross frequency (BCF) [33]. The pH of each sample was analyzed using a Medidor PH BASIC 20 (Hach Large srl, Milan, Italy) after calibration according to the manufacturer’s instructions. 

### 4.5. Scanning Electron Microscopy 

Observations by Scanning Electron Microscopy (SEM) were performed on samples treated with the lowest, middle, and highest concentrations of TTO (0.2 mg/mL; 1 mg/mL; 2 mg/mL) and TER (0.08 mg/mL; 0.42 mg/mL; 0.83 mg/mL) to visualize, if present, membrane morphological alterations.

Aliquots of 500 μL from the above-mentioned samples were centrifuged at 800× *g* for 10 min, and the pellet fixed in 500 μL of 5% glutaraldehyde solution buffered at pH 7.2 with phosphate buffer 0.1 M. The samples were resuspended and a drop of each was pipetted on a stapled filter paper bag and washed with phosphate buffer 0.1 M pH 7.2. Dehydration was performed by means of increasing concentrations of aqueous ethanol (10%, 20%, 30%, 50%, 75%, and 95%) for 15 min and in 100% ethanol for 5 min at 5 °C. Specimens were then dried with a critical point drier unit K850 (Emitech Ltd., Ashford, UK), mounted on aluminum stubs with double stick tape and coated with a gold–palladium film using an ion sputtering unit K500 (Emitech Ltd., Ashford, UK). The dry samples were then observed with a 515 SEM (Philips, Eindhoven, NL) at 10 kV, and the pictures were taken with a 5400 Coolpix digital camera (Nikon, Chiyoda-ku, Tokyo, Japan).

### 4.6. Statistical Analyses

The statistical analyses were performed using the software R 3.0.3 (The R Foundation for Statistical Computing) and graphically represented using the software GraphPad Prism v.8 (GraphPad Software Inc., San Diego, CA, USA). Descriptive statistics were calculated and expressed as means and standard error of the mean. Normal distribution was assessed by means of the Shapiro-Wilk test (*p* < 0.05). To evaluate differences between the treated samples and the control one, a one-way ANOVA followed by the Dunnett post-hoc test was performed (significance level set at 0.05). Linear regression models were set up to analyze the potential concentration-dependent effects.

## 5. Conclusions

In conclusion, this study highlights the importance of evaluating the effects of natural compounds on spermatozoa before suggesting and exploiting their beneficial effects in the reproductive field. As already reported, the analytical approach seems to provide robust and repeatable results that may also help to unveil the mechanism of interactions of such compounds. Motility proved to be the most sensitive parameter for the analysis of tea tree oil, strengthening the idea of future applications of spermatozoa in toxicological screenings, in the light of their mitochondria abundance and their capability to generate movement. The concentrations of TTO and TER that resulted as non-toxic on the basis of this study need further investigations for potential exploitation in the reproductive field.

## Figures and Tables

**Table 1:** Composition of tea tree oil (TTO).

Compound	LRI ^1^	Area%
terpinen-4-ol	1185	41.49
γ-terpinene	1061	20.55
α-terpinene	1018	9.59
α-terpineol	1194	4.42
α-pinene	933	4.4
*p*-cymene	1025	3.66
terpinolene	1089	3.18
1,8-cineole	1031	2.15
limonene	1029	1.78
aromadendrene	1446	1.38
caryophyllene oxide	1594	0.76
myrcene	992	0.72
allo-aromadendrene	1468	0.18
α-felladrene	1005	0.17
sabinene	973	0.03
β-pinene	975	0.03
α-humulene	1460	0.02
β-caryophyllene	1425	0.02
Total		94.53

^1^ LRI = linear retention index.

**Table 2:** Effects of tea tree oil (TTO) on sperm kinematic parameters for samples with total motility ≥20%. Data are reported as Mean (standard error of the mean), *n* = 9. Differences were calculated by means of Dunnett PostHoc test (* = *p* < 0.05; ** = *p* < 0.01; *** = *p* < 0.001).

	TTO (mg/mL)					
	CTR	0.2	0.4	0.6	0.8	1
VAP (μm/s)	80.96 (2.63)	77.70 (3.89)	81.02 (5.63)	79.04 (4.37)	72.47 (3.15)	54.95 (7.87) ***
VCL (μm/s)	180.75 (5.62)	173.34 (8.61)	186.84 (12.73)	180.77 (6.22)	165.14 (6.14)	132.98 (16.83) **
VSL (μm/s)	41.10 (1.73)	37.30 (2.00)	34.35 (2.29)	33.33 (2.19)	32.02 (1.27)	25.36 (2.16) ***
DAP (μm)	48.19 (1.39)	47.37 (1.89)	49.14 (2.77)	48.08 (2.41)	44.78 (2.11)	36.71 (4.19) **
DCL (μm)	110.20 (3.49)	106.94 (4.83)	115.65 (6.59)	112.55 (3.62)	104.97 (5.06)	86.34 (9.86) *
DSL (μm)	22.71 (0.79)	20.88 (0.96)	18.45 (0.94) *	18.27 (1.12) *	18.11 (0.32) *	15.24 (0.83) ***
LIN (%)	22.74 (0.60)	22.58 (0.55)	19.54 (0.86)	20.07 (1.32)	21.03 (1.25)	21.41 (1.60)
STR (%)	50.90 (1.09)	48.67 (0.97)	43.19 (1.28) **	43.37 (2.16) *	46.48 (2.42)	47.43 (3.03)
WOB (%)	45.16 (0.32)	45.00 (0.35)	43.64 (0.89)	44.48 (1.02)	44.06 (0.83)	43.96 (0.97)
ALH (μm)	9.50 (0.26)	8.88 (0.38)	9.55 (0.69)	10.01 (0.44)	9.54 (0.66)	8.65 (1.01)
BCF(Hz)	37.75 (1.15)	37.34 (0.77)	38.15 (0.99)	36.60 (2.89)	34.94 (1.38)	35.11 (2.75)

VAP = velocity average path; VCL = velocity curved line; VSL = velocity straight line; DAP = distance average path: DCL = distance curved line; DSL = distance straight line; LIN = linearity (VSL/VCL); STR = straightness (VSL/VAP); WOB = wobble (VAP/VCL); ALH = amplitude of lateral head displacement; BCF = beat cross frequency.

**Table 3:** Effects of Terpinen-4-ol (TER) on sperm kinematic parameters for samples with total motility ≥20%. Data are reported as Mean (standard error of the mean), *n* = 9. Differences were calculated by means of Dunnett PostHoc test (* = *p* < 0.05; ** = *p* < 0.01; *** = *p* < 0.001).

	TER (mg/mL)										
	CTR	0.08	0.17	0.25	0.33	0.42	0.50	0.58	0.67	0.75	0.83
	Equivalent to TTO (mg/mL)										
		0.2	0.4	0.6	0.8	1	1.2	1.4	1.6	1.8	2
VAP (µm/s)	88.12 (2.26)	92.15 (4.46)	92.29 (4.22)	95.19 (5.92)	90.17 (5.92)	86.83 (6.27)	87.91 (5.09)	87.89 (5.61)	78.58 (5.71)	75.39 (6.40)	75.41 (4.92)
VCL (µm/s)	200.47 (5.82)	212.65 (12.22)	219.15 (7.81)	223.22 (8.89)	212.76 (9.48)	200.71 (12.55)	202.52 (11.31)	203.59 (11.60)	188.61 (13.59)	172.23 (12.16)	170.46 (10.83)
VSL (µm/s)	44.32 (1.63)	44.77 (1.56)	36.71 (3.73)	37.25 (1.88)	36.68 (3.25)	34.88 (2.86) *	35.54 (2.87) **	33.78 (1.90) **	27.72 (2.33) ***	28.15 (1.97) ***	29.19 (2.61) ***
DAP (µm)	51.32 (1.53)	54.01 (2.78)	54.23 (2.78)	55.39 (3.32)	53.45 (3.73)	51.58 (3.34)	51.15 (3.42)	50.77 (4.66)	48.48 (3.01)	44.37 (4.45)	45.79 (2.48)
DCL (µm)	119.94 (3.40)	127.73 (8.35)	132.63 (3.80)	134.08 (5.57)	129.96 (7.87)	123.33 (6.62)	122.08 (7.39)	124.67 (7.34)	121.17 (7.45)	108.95 (6.67)	107.95 (6.26)
DSL (µm)	23.62 (1.31)	24.27 (0.98)	19.08 (2.18)	19.36 (1.33)	19.28 (0.99)	18.06 (0.82) *	18.25 (0.94) *	17.88 (1.10) *	15.32 (1.25) ***	15.24 (1.10) ***	16.13 (1.41) ***
LIN (%)	22.93 (1.28)	22.67 (1.68)	18.15 (1.81)	18.16 (1.25)	18.74 (1.42)	20.09 (1.35)	17.03 (1.15) *	17.47 (0.50) *	15.95 (0.66) *	16.66 (0.69) *	16.23 (1.81) **
STR (%)	49.45 (2.11)	48.83 (2.65)	41.79 (3.25)	41.04 (2.60)	41.66 (2.51)	41.52 (1.71)	41.32 (0.91)	39.64 (1.20) *	36.99 (1.48) **	38.67 (2.19) **	39.01 (1.62) **
WOB (%)	10.31 (0.30)	10.57 (0.59)	174.74 (0.36)	11.38 (0.34)	11.08 (0.52)	10.95 (0.37)	43.55 (0.37)	42.98 (0.68)	42.28 (0.78)	43.54 (0.81)	44.29 (1.16)
ALH (µm)	10.31 (0.30)	10.57 (0.59)	11.25 (0.39)	11.38 (0.36)	33.08 (0.34)	10.95 (0.52)	11.44 (0.42)	11.30 (0.51)	12.34 (1.59)	10.67 (0.58)	10.67 (0.39)
BCF (Hz)	45.05 (3.30)	43.52 (3.95)	48.68 (7.60)	42.48 (4.63)	41.13 (5.56)	36.21 (1.82)	32.47 (1.01)	36.27 (1.82)	40.78 (5.74)	34.56 (2.00)	33.38 (2.29)

VAP = velocity average path; VCL = velocity curved line; VSL = velocity straight line; DAP = distance average path: DCL = distance curved line; DSL = distance straight line; LIN = linearity (VSL/VCL); STR = straightness (VSL/VAP); WOB = wobble (VAP/VCL); ALH = amplitude of lateral head displacement; BCF = beat cross frequency.

**Table 4:** Simple linear regression models’ angular coefficients (*β*).

	Tea Tree Oil	Terpinen-4-ol
	*β* (C.I. 95%)	*β* (C.I. 95%)
V %	−0.091 (−0.104; −0.079) *p* < 0.0001	0.094 (0.010; 0.178) *p* = 0.0279
TotM %	−0.080 (−0.090: −0.070) *p* < 0.0001	0.049 (0.008; 0.091) *p* = 0.020
ProgM %	−0.163 (−0.185; −0.141) *p* < 0.0001	0.017 (−0.043; 0.077) *p* =0.564
AR %	0.100 (0.086; 0.114) *p* < 0.0001	−0.089 (−0.237; 0.060) *p* = 0.239
pH value	−2.071 (−10.308; 6.166) *p* = 0.619	4.348 (−8.203; 16.899) *p* = 0.491

C.I. = Confidence Interval; V = Viability; TotM = Total Motility; ProgM = Progressive Motility; AR = Acrosome Reaction.

**Table 5:** Concentrations of tea tree oil (TTO) and Terpinen-4-ol (TER) used in the study. TER concentrations were calculated according to its presence in the used TTO (41.5%).

TTO (mg/mL)	TER (41.5 %) (mg/mL)
2	0.83
1.8	0.75
1.6	0.67
1.4	0.58
1.2	0.5
1	0.42
0.8	0.33
0.6	0.25
0.4	0.17
0.2	0.08

## Supplementary Materials

The Supplementary Materials are available online. Table S1: descriptive statistics of the effects of tea tree oil on semen morpho-functional parameters, Table S2: descriptive statistics of the effects of terpinen-4-ol on semen morpho-functional parameters.
