# Supplementary material for: In Vitro Effects of Tea Tree Oil (Melaleuca Alternifolia Essential Oil) and its Principal Component Terpinen-4-ol on Swine Spermatozoa
Source: Molecules. 2019 Mar 19;24(6):1071. doi: 10.3390/molecules24061071 (PMC6471158; doi:10.3390/molecules24061071)
Supplement: Supplementary file 1 [file molecules-24-01071-s001.pdf]

Supplementary file

In vitro effects of tea tree oil (*Melaleuca alternifolia* essential oil) and its principal component terpinen-4-ol on swine spermatozoa

Alberto Elmi, Domenico Ventrella, Barone Francesca, Giacomo Carnevali, Gianfranco Filippini, Annamaria Pisi, Stefania Benvenuti, Maurizio Scozzoli, Maria Laura Bacci

Table S1. Descriptive statistics of the effects of tea tree oil on semen morpho-functional parameters.

Data are reported as Mean (standard error of the mean). n=9.

	**TTO (mg/mL)**										
	**CTR**	**0.2**	**0.4**	**0.6**	**0.8**	**1**	**1.2**	**1.4**	**1.6**	**1.8**	**2**
**V (%)**	89.00	91.43	87.71	84.33	78.75	68.92	67.71	43.33	22.00	19.25	9.00
	(1.03)	(1.32)	(2.32)	(2.34)	(4.12)	(7.20)	(7.05)	(11.53)	(8.93)	(9.36)	(5.13)
**TotM.(%)**	78.38	77.76	74.92	65.88	56.48	43.04	14.79	3.03	1.00	0.71	0.86
	(1.46)	(3.19)	(4.99)	(8.94)	(11.81)	(11.09)	(7.02)	(2.29)	(0.20)	(0.07)	(0.21)
**ProgM (%)**	38.00	31.93	23.39	20.71	15.51	11.28	4.18	0.24	0.03	0.00	0.00
	(2.60)	(3.77)	(10.42)	(4.93)	(5.65)	(3.65)	(2.88)	(0.21)	(0.03)	(0.00)	(0.00)
**AR (%)**	2.83	3.13	3.64	5.44	9.36	10.42	18.00	44.25	56.06	68.31	68.63
	(0.39)	(0.51)	(0.92)	(0.85)	(1.78)	(1.73)	(3.97)	(6.43)	(7.42)	(8.24)	(9.44)
**pH**	6.69	6.68	6.67	6.72	6.68	6.68	6.68	6.69	6.68	6.67	6.69
	(0.01)	(0.03)	(0.03)	(0.05)	(0.03)	(0.02)	(0.02)	(0.02)	(0.03)	(0.03)	(0.04)

TTO= tea tree oil; V= Viability; TotM= Total Motility; ProgM= Progressive Motility; AR= Acrosome Reaction

Table S2. Descriptive statistics of the effects of terpinen-4-ol on semen morpho-functional parameters.

Data are reported as Mean (standard error of the mean), n=9.

	TER (mg/mL)										
	**CTR**	**0.08**	**0.17**	**0.25**	**0.33**	**0.42**	**0.5**	**0.58**	**0.67**	**0.75**	**0.83**
	equivalent to TTO (mg/mL)										
		0.2	0.4	0.6	0.8	1	1.2	1.4	1.6	1.8	2
**V (%)**	90.09	90.50	90.21	89.86	88.94	87.62	86.31	84.19	80.88	78.31	75.56
	(1.07)	(2.24)	(2.33)	(1.62)	(2.11)	(2.23)	(1.57)	(2.31)	(2.22)	(2.87)	(3.10)
**TotM (%)**	80.19	81.98	83.76	82.14	85.72	80.67	75.94	78.10	65.77	62.94	60.29
	(1.82)	(2.74)	(3.12)	(3.32)	(2.26)	(4.60)	(5.17)	(4.01)	(7.89)	(9.02)	(9.09)
**ProgM (%)**	40.94	39.52	34.52	27.46	31.75	27.98	24.77	24.20	17.14	16.80	17.04
	(2.64)	(3.62)	(4.69)	(2.53)	(3.96)	(3.38)	(2.47)	(1.79)	(3.13)	(2.99)	(4.01)
**AR (%)**	4.05	4.08	4.92	5.58	6.64	6.67	8.86	9.07	11.00	12.29	11.14
	(0.32)	(0.40)	(0.58)	(0.86)	(1.11)	(0.91)	(1.62)	(1.54)	(1.99)	(3.39)	(2.13)
**pH**	6.72	6.70	6.71	6.71	6.73	6.72	6.69	6.69	6.67	6.67	6.66
	(0.02)	(0.40)	(0.02)	(0.03)	(0.05)	(0.04)	(0.02)	(0.03)	(0.01)	(0.01)	(0.01)

TER= terpinen-4-ol; V= Viability; TotM= Total Motility; ProgM= Progressive Motility; AR= Acrosome Reaction
